# Targeting SUMOylation in glioblastoma: A novel avenue for therapy and biomarker discovery

**DOI:** 10.1016/j.gendis.2025.101841

**Published:** 2025-09-02

**Authors:** Wiktoria Dubanosow, Bartosz Lenda, Marta Żebrowska-Nawrocka, Dagmara Szmajda-Krygier, Rafał Świechowski, Ewa Balcerczak

**Affiliations:** Laboratory of Molecular Diagnostics, Department of Pharmaceutical Biochemistry and Molecular Diagnostics, BRaIN Laboratories, Medical University of Lodz, Czechoslowacka 4, Lodz 92-216, Poland

**Keywords:** Cell cycle, Glioblastoma, Post translational modifications, SAE1, SUMOylation

## Abstract

SUMOylation, a post-translational protein modification, plays a crucial role in regulating various biological processes. Dysregulation of SUMOylation has been linked to glioblastoma progression, impacting key signaling pathways. This review summarizes the current knowledge on SUMOylation's role in glioma malignancy, highlighting its influence on cell cycle regulation, PKB/AKT signaling pathway, and microRNA expression. Our work identifies Ubc9 as a promising therapeutic target due to its role in enhancing SUMOylation, promoting glioblastoma aggressiveness, and facilitating tumor proliferation. Additionally, SAE1 correlates with glioblastoma grade and affects cell cycle regulators, while SUMOylation stabilizes CDK6, driving the G1/S transition. Targeting these pathways with inhibitors, such as topotecan and chlorogenic acid, may provide novel treatment strategies. Furthermore, SUMOylation-driven alterations in transcription factors and DNA repair mechanisms contribute to therapy resistance. Understanding these mechanisms could pave the way for innovative interventions in glioblastoma management.

## Introduction

Gliomas are the most common primary tumors of the central nervous system in the adult population. They are characterized by highly infiltrative, rapid, and aggressive growth.^1^ The most frequently occurring glioma among adults is glioblastoma (GBM), which is classified as a grade IV malignancy according to the latest WHO classification of central nervous system tumors.^2^^,^^3^ Despite the use of traditional treatments, including surgical resection combined with chemo- and/or radiotherapy, the median survival is approximately 15 months. In recent years, GBM has gained attention among researchers due to advances in molecular biology, enabling detailed studies of the tumor's genetic profile and offering hope for newly diagnosed patients through personalized treatment.^4^ Currently, standard practice includes the detection of isocitrate dehydrogenase (*IDH*) gene mutations, which allows the classification of patients into an appropriate group and facilitates the selection of optimal treatment.^5^

Development of gliomas is closely linked to disruptions in the cell cycle, which arises from abnormalities in post-translational protein modifications (PTMs) affecting the regulation of cellular processes (*e.g.*, cell proliferation and apoptosis). The cell cycle is a kinase-dependent process that orchestrates the duplication of genetic material and its division into two genetically identical daughter cells. It consists of four main stages: G1 phase, which prepares the cell for division; S phase, where genetic material is duplicated; G2 phase, which ensures the integrity of the duplicated genetic material; and the M phase, during which cell division occurs. Additionally, certain cells may exit the cycle and enter the G0 resting phase.^6^ Understanding the molecular disruptions underlying glioma development requires a detailed exploration of the cell cycle and its regulation, as this process is a central target of oncogenic alterations in gliomas.^7^

Precise regulation of the cell cycle is critical for maintaining genomic stability and proper cellular function. This regulation is mediated by intricate control mechanisms, many of which depend on PTMs. These biochemical modifications are essential for maintaining cell cycle fidelity by modulating protein stability, DNA repair mechanisms, and transcriptional regulation. Emerging evidence suggests that PTMs, including phosphorylation, ubiquitination, and methylation, may play a pivotal role in driving oncogenic transformation and the invasive properties characteristic of many human neoplasms.^8^, ^9^, ^10^ Aberrations in these processes are frequently observed in gliomas, contributing not only to uncontrolled proliferation but also to the tumor's invasive and aggressive behavior, highlighting their critical role in glioma progression. Moreover, they can contribute to therapy resistance, underscoring their significance in the tumor microenvironment. Identifying and targeting these molecular aberrations hold promise for advancing therapeutic strategies and improving clinical outcomes for patients with gliomas.^11^

Ubiquitination and SUMOylation are similar processes and contribute to protein regulation. Small ubiquitin-like modifier (SUMO), responsible for SUMOylation, and ubiquitin, responsible for ubiquitination, are nearly the same in molecular structure, but due to small differences in amino acid sequence, they play distinct roles in PTMs. Ubiquitin is usually involved in degrading proteins via the proteasome (programmed cell death). SUMOylation plays a role in regulating protein subcellular localization, protein–DNA binding, protein–protein interactions, transcriptional regulation, DNA repair, and genome organization without triggering protein degradation.^11^ Both processes are necessary for the proper development of all organisms, but any changes in the physiological functioning of ubiquitination and SUMOylation can lead to pathology. New studies emphasize the importance of SUMOylation in various diseases, such as liver diseases,^12^ neurodegenerative diseases,^13^ and cancers.^14^

Due to the poor overall survival rate and limited treatment options and diagnostic methods, it is important to find new molecular markers, which could be helpful for clinical practice in glioma management. Currently, PTMs, including SUMOylation, are gaining increasing attention due to the growing body of research on their impact on glioma progression. The connection between cell cycle, SUMOylation, and glioma progression seems to be a promising perspective not only for diagnostic aspects. A good understanding of the molecular pathways connecting the cell cycle, glioma development, and SUMOylation may represent a promising perspective as a new therapeutic option. Therefore, it is essential to thoroughly investigate the aforementioned relationships.

In this work, we decided to focus on well-known signaling pathways with an emphasis on SUMO-related processes in high-grade gliomas. Additionally, we reviewed the role of SUMO-targeting drugs in cancer and glioma *in vitro* and *in vivo* models. Considering the rapid progress in the field and the growing number of studies on central nervous system tumors, there is a high probability of a potential breakthrough in this subject.

### SUMO

SUMOs are small proteins of approximately 100 amino acids and share about 18% homology to ubiquitin. In mammals, there are five isoforms of SUMO protein: SUMO1, SUMO2, SUMO3, SUMO4, and SUMO5. SUMO2 and SUMO3 are similar in amino acid sequence (about 95% homology; they are often referred to as SUMO2/3 due to this similarity), but SUMO1 has 50% homology with SUMO2/3 and is remarkably less expressed than SUMO2, which is the most expressed SUMO protein in human tissues.^15^ SUMO4 shares similarities with SUMO2/3, but it differs in its sequence features: a proline at position 90 instead of a glutamine. SUMO1, SUMO2, and SUMO3 are expressed in all human organs. SUMO4 mRNA is only expressed in the spleen, lymph nodes, and kidneys. SUMO5 is a newly discovered SUMO, and its presence was detected in testes and peripheral hemolymph.^16^

**Figure 1:**
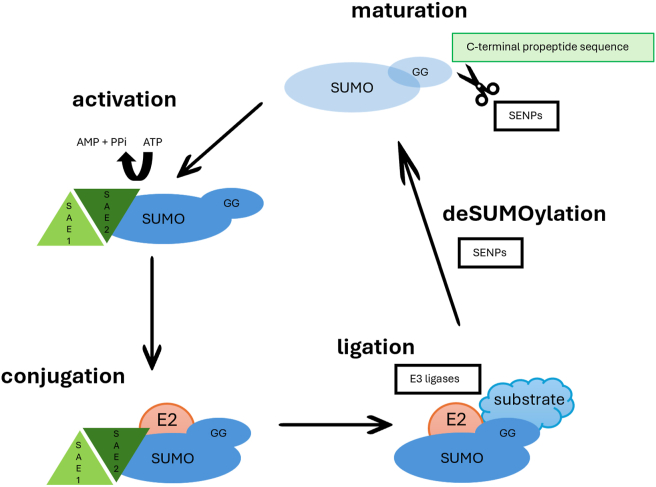
The SUMO catalytic cycle. The diagram illustrates the SUMOylation pathway, including its key steps: maturation, activation, conjugation, ligation, and de-modification. The process begins with SUMO precursors undergoing maturation by SUMO-specific proteases (SENPs). Activated SUMO proteins are transferred to the E1 enzyme (SAE1-SAE2 heterodimer) in an ATP-dependent process. Subsequently, the activated SUMO is handed over to the E2 enzyme (Ubc9) through conjugation. E3 ligases then facilitate the transfer of SUMO from Ubc9 to the lysine residue of target proteins, forming an isopeptide bond. Finally, SENPs catalyze deSUMOylation to reverse the modification, ensuring dynamic regulation of protein function.^25^

SUMO cycle is a catalytic, reversible process, which consists of five phases: maturation, activation, conjugation, ligation, and deSUMOylation (Fig. 1). The whole process is under the control of an enzymatic cascade. After translation, all SUMO proteins weigh about 11 kDa and are inactive proteins (precursor forms). They contain a C-terminal propeptide sequence. This additional sequence prevents their immediate conjugation to target proteins and must be removed to expose the conserved diglycine (-GG) motif, which is essential for the subsequent steps of SUMOylation. This whole process is called maturation and is under the control of SUMO-specific proteases, known as sentrin-specific proteases (SENPs). In mammals, the primary enzymes responsible for SUMO maturation are SENP1 and SENP2. Those enzymes remove four amino acids from the C-terminal, exposing a diglycine (GG) motif, enabling the next stage, which is the activation of the SUMO protein.

The enzyme responsible for SUMO protein activation is the E1-activating enzyme, which consists of two subunits, SUMO-activating enzyme 1 (SAE1 or Aos1) and SUMO-activating enzyme 2 (SAE2 or UBA2). Those two subunits typically assemble into a heterodimer, either as SAE1-SAE2 or Aos1-UBA2. This heterodimer facilitates the activation of the SUMO protein via ATP-dependent SUMO adenine nucleotide intermediates, forming a thioester bond between the C-terminal carboxyl group of SUMO and the cysteine residue of SAE2/UBA2.^16^

The conjugation process involves the transfer of the activated SUMO protein to the cysteine residue at position 93 of Ubc9 (also known as ubiquitin-conjugating enzyme E2 (UBE2I)), the sole E2 enzyme responsible for SUMO conjugation in the SUMOylation pathway. E2 interacts with the E1-SUMO intermediate by binding to SAE2 through a transesterification reaction, resulting in the formation of an E2-SUMO thioester complex. Once bound, SUMO is transferred from the E1-SUMO intermediate to the catalytic cysteine residue (C93) of Ubc9 through a transesterification reaction, forming a thioester bond. Ubc9 then catalyzes the conjugation of SUMO to the target protein (see Table 1 for examples of SUMOylated substrates) by forming an isopeptide bond with the lysine residue of the substrate. Importantly, Ubc9 plays a critical role in substrate recognition by directly interacting with a SUMO consensus motif (ΨKxE/D, where Ψ represents a large hydrophobic residue) surrounding the lysine residue of the target protein. This ensures substrate specificity and precise SUMOylation.^16^^,^^17^

**Table 1:** Examples of SUMO target proteins and the potential SUMOylation influence on tumor progression.

Target protein	SUMOylation	Reference
Rb	SUMOylation site: Rb is SUMOylated primarily at lysine residues. Function: SUMOylation of Rb influences its ability to regulate the cell cycle, gene transcription, and tumor suppression. Effects: Enhances Rb's repressive function on E2F transcription factors, leading to cell cycle arrest; affects chromatin remodeling and gene silencing; modulates interactions with other regulatory proteins (*e.g.*, p53).	Chen et al^26^
NF-κB	SUMOylation site: NF-κB subunits, such as RelA (p65), are SUMOylated at specific lysine residues (*e.g.*, K310 in p65). Function: SUMOylation fine-tunes NF-κB signaling, balancing pro-inflammatory and anti-inflammatory responses. Effects: Suppresses NF-κB transcriptional activity, leading to reduced expression of inflammatory genes; enhances recruitment of corepressors, limiting excessive immune activation; modulates crosstalk with other post-translational modifications (*e.g.*, ubiquitination and phosphorylation); affects NF-κB-dependent apoptosis and stress responses.	Liu et al^27^
p53	SUMOylation site: Lysine 386 (K386). Function: SUMOylation regulates p53's activity as a tumor suppressor, influencing its localization, stability, and transcriptional activity. Effects: Modulates p53-dependent gene expression; enhances transcriptional repression of specific target genes; influences DNA damage response and apoptosis regulation; can either stabilize or destabilize p53, depending on cellular context.	Chen et al^26^ Wu et al^28^
Olig2	SUMOylation site: K27, K76, K112 (lysine residues). Function: Inhibition of p53-mediated cell cycle arrest, promotion of tumor growth, and glioblastoma chemoresistance. Effects: Suppress p53-induced apoptosis; promotes tumor proliferation; induces chemoresistance to temozolomid (glioblastoma).	Liu et al^29^ Ligon et al^30^
Mdm2	SUMOylation site: Lysine 182 (K182), Lysine 185 (K185). Function: SUMOylation enhances Mdm2 stability and modulates its E3 ubiquitin ligase activity. Effects: Increases Mdm2 stability by preventing its proteasomal degradation; regulates nuclear localization; modulates Mdm2-mediated ubiquitination of p53, affecting p53-dependent apoptosis and cell cycle control; contributes to tumorigenesis by promoting p53 inhibition.	Miyauchi et al^31^ Chen et al^32^
Vimentin	SUMOylation site: Vimentin undergoes SUMOylation at lysine residues K354, K466, and K514. Function: SUMOylation of vimentin influences its role in maintaining cell shape, motility, and interactions with other proteins. Effects: Inhibition of cell motility reduces tumor invasiveness; interaction with PIAS3 affects its functional properties, potentially impacting glioma progression.	Wang et al^33^
SOX2	SUMOylation site: Specific lysine residues within SOX2 that undergo SUMOylation have not been conclusively identified. Function: SUMOylation of SOX2 is believed to modulate its transcriptional activity, influencing the expression of genes involved in stem cell maintenance and tumorigenesis. Effects: Stem cell maintenance: SOX2 is crucial for maintaining the stem-like properties of glioma stem cells, including self-renewal and pluripotency; enhances tumorigenic potential, promoting proliferation and survival of glioma cells; enhances invasive and migratory capabilities of cells, contributing to tumor progression and metastasis.	Garros-Regulez et al^34^
PML	SUMOylation site: Lysine 160 (K160). Function: SUMOylation of PML is crucial for the formation and maintenance of PML nuclear bodies (PML-Körper), which play a key role in regulating processes, such as transcription, DNA repair, and cellular stress response. Effects: Enhances the recruitment of other proteins to PML nuclear bodies; modulates the function of recruited proteins; increases acetylation, SUMOylation, and phosphorylation of p53; strengthens p53's tumor suppressor activity; regulates apoptosis and cell cycle control.	Zhang et al^35^
hnRNP A2/B1	SUMOylation site: Arginine 108 (R108). Function: SUMOylation at R108 facilitates the translocation of hnRNP A2/B1 from the nucleus to the cytoplasm under hypoxic conditions. Effects: Promotes the export of miR-204-3p into exosomes, leading to its depletion in glioma cells; reduces the tumor-suppressive effects of miR-204-3p, thereby enhancing angiogenesis and tumor growth; inhibition of hnRNP A2/B1 SUMOylation impedes these processes, suggesting potential therapeutic avenues.	Guo et al^36^
AKT	SUMOylation site: Lysine 14 (K14). Function: SUMOylation at K14 modulates AKT stability and activity, influencing its role in cell signaling. Effects: Increases AKT stability; enhances PI3K/AKT signaling pathway; promotes uncontrolled cell growth, migration, and angiogenesis in gliomas; contributes to tumor development and progression.	Yang et al^23^
CDK6	SUMOylation site: Lysine residues (exact sites are not definitively identified in available sources). Function: SUMOylation of CDK6 leads to its stabilization, facilitating the progression of the cell cycle from G1 to S phase in glioma cells. Effects: Increases CDK6 stability; accelerates cell cycle progression; enhances glioma cell proliferation; potential contribution to tumor development and progression.	Bellali et al^37^
UBE2I (Ubc9)	SUMOylation site: UBE2I itself is SUMOylated at specific lysine residues, though the exact positions are not detailed in the current literature. Function: SUMOylation of UBE2I enhances its isomerization activity, which in turn promotes the SUMOylation of various target proteins. Effects: Promotes PUM2 SUMOylation (increased invasiveness).	Wang et al^38^
UBA2 (SAE2)	SUMOylation site: UBA2 itself is SUMOylated at specific lysine residues, but the exact positions are not well defined in the literature. Function: SUMOylation of UBA2 enhances its activity, promoting the SUMOylation of various target proteins. Effects: Increases cell proliferation, migration, invasion, and vasculogenic mimicry; increases aggressiveness and poor prognosis in glioma patients.	Cao et al^39^
SUMO1	SUMOylation site: SUMO1 attaches to specific lysine residues on target proteins; however, the exact sites vary depending on the substrate. Function: SUMO1 modification regulates multiple cellular processes, including nuclear-cytoplasmic transport, transcriptional regulation, apoptosis, and DNA repair. Effects: Cell cycle regulation: uncontrolled cell proliferation (*e.g.*, in glioblastoma); increased malignancy.	Bellail et al^37^
HIF-1α	SUMOylation site: HIF-1α undergoes SUMOylation at specific lysine residues; however, the precise sites are not well-characterized in the current literature. Function: SUMOylation of HIF-1α influences its stability and activity. Effects: Stabilizes HIF-1α, promoting angiogenesis and invasion; affects the migration of Tregs, thereby modulating immune responses.	Kaur et al^40^
PUM2	SUMOylation site: Specific lysine residues within PUM2 that undergo SUMOylation in gliomas have not been conclusively identified. Function: SUMOylation of PUM2 influences its stability and interaction with other proteins, thereby modulating its role in regulating gene expression and cellular processes. Effects: Enhances its interaction with CEBPD, leading to increased expression of DSG2, which promotes vasculogenic mimicry, a blood supply channel distinct from endothelial blood vessels; tumor progression and metastasis.	Wang et al^38^
STAT3	SUMOylation site: SUMOylation of STAT3 occurs at lysine 685. Function: SUMOylation of STAT3 modulates its transcriptional activity and stability. Effects: Cell survival, inflammation, and proliferation contribute to head and neck neoplasm progression.	Zhou et al^41^
*c-Myc*	SUMOylation site: c-Myc undergoes SUMOylation at lysine residues, notably at lysine 51 (K51). This modification is facilitated by SUMO E3 ligases, such as PIAS1. Function: SUMOylation of c-Myc affects its transcriptional activity and stability by repressing its ability to activate target gene transcription and altering its interaction with co-factors and DNA. It can also promote ubiquitin-mediated degradation of c-Myc, reducing its stability. Effects: Represses c-Myc transcriptional activity, affecting glioma-related gene expression; Marks c-Myc for degradation, decreasing its protein levels; potentially inhibits glioma cell proliferation through altered c-Myc function; modulating SUMOylation of c-Myc may serve as a strategy for glioma treatment.	Suna et al^42^

**Note:** Rb, retinoblastoma protein; NF-κB, nuclear factor kappa B; p53, tumor protein p53; Olig2, oligodendrocyte transcription factor 2; Mdm2, murine double minute 2; SOX2, SRY-box transcription factor 2; PML, promyelocytic leukemia protein; hnRNP A2/B1, heterogeneous nuclear ribonucleoprotein A2/B1; AKT, protein kinase B; CDK6, cyclin-dependent kinase 6; UBE2I, ubiquitin-conjugating enzyme E2I; UBA2, SUMO-activating enzyme 2; SUMO1, small ubiquitin-like modifier 1; HIF-1α, hypoxia-inducible factor 1 alpha; PUM2, pumilio RNA-binding family member 2; c-Myc, cellular myelocytomatosis oncogene; STAT3, signal transducer and activator of transcription 3; **RelA,***v-rel avian reticuloendotheliosis viral oncogene homolog A*; Tregs, regulatory T cells; CEBPD, CCAAT/enhancer-binding protein delta; DSG2, desmoglein-2.

While SUMO E1 and Ubc9 alone can SUMOylate substrates, SUMO E3 ligases are crucial for efficient substrate targeting. Examples of SUMO E3 ligases are PIAS (protein inhibitor of activated STAT) family, which regulates transcription factors and signaling pathways, Ran-binding protein 2 (RanBP2), which is involved in nucleocytoplasmatic transport, and zinc finger protein 451 (ZNF451), which functions as an E3 ligase with a unique dual SUMOylation mechanism, allowing it to catalyze the conjugation of two SUMO molecules in a single step.^18^ These ligases enhance ligation by stabilizing the interaction between the substrate and the SUMO-E2 complex. They increase SUMOylation efficiency by positioning the substrate optimally for SUMO transfer from Ubc9, reducing the energy barrier for conjugation. By precisely positioning the complex, SUMO E3 ligases reduce the distance between the SUMO-E2 thioester bond and the substrate's lysine residue, increasing both specificity and efficiency of the process.^19^^,^^20^

DeSUMOylation is the process of removing SUMO molecules from target proteins, allowing for the dynamic regulation of protein function. This part of the SUMO catalytic cycle is catalyzed by specific SUMO proteases (SENPs), which act as isopeptidases by hydrolyzing isopeptide bonds between SUMO and the lysine residue of the substrate protein. In humans, there are six main SENP proteases with different specificities. SENP1 and SENP2 exhibit specificity for SUMO1 and SUMO2/3, playing crucial roles in deSUMOylation of nuclear and cytoplasmic proteins. They regulate transcriptional pathways and stress responses by reversing SUMO modifications, thereby modulating protein activity and stability. SENP3 and SENP5 exhibit specificity for SUMO2/3 and primarily function in the nucleolus. They regulate ribosome biogenesis, mitochondrial dynamics, and cellular stress responses by removing SUMO modifications from target proteins. SENP6 and SENP7 preferentially process SUMO2/3 chains, acting as SUMO deconjugating enzymes that prevent polySUMO chain accumulation. They play key roles in genome stability, DNA damage repair, and inflammatory signaling by maintaining balanced SUMOylation levels. These proteases dynamically regulate the balance between SUMO conjugation and deSUMOylation, fine-tuning protein localization, stability, and activity in response to cellular cues.^15^^,^^16^^,^^21^^,^^22^ DeSUMOylation has three stages: the first stage is substrate recognition: SENP proteases identify SUMOylated proteins through interaction with SUMO regions and modification sites on target proteins; SENPs then catalyze the breakdown of the bound between SUMO and the lysine of the substrate, releasing SUMO for reuse; the last step is SUMO recycling: after detaching from the substrate, free SUMO can participate again in the SUMOylation process, ensuring dynamic control of cellular proteins. Figure 1 illustrates the SUMOylation process in a graphical form.

Dysregulation of SUMOylation enzyme expression is associated with the occurrence of cancers and patients' prognosis. For example, bioinformatic analyses show that increased expression of SAE1 and SAE2, which are components of E1, among others, correlates with decreased survival rates and accelerated tumor progression in glioma patients.^23^^,^^24^

### Selectivity and dynamics of SUMOylation

SUMOylation is a highly dynamic and selective post-translational process that modulates protein activity, localization, and stability. SUMO1 and SUMO2/3 differ in function and localization: SUMO1 is primarily conjugated under steady-state conditions and is mainly found in the nucleoplasm, where it is associated with nuclear structures such as promyelocytic leukemia nuclear bodies. In contrast, SUMO2/3 is present in the nucleus and cytoplasm and has even been detected in the axon.^43^^,^^44^ The varied distribution of SUMO isoforms influences their roles in maintaining nuclear architecture, responding to stress, and supporting neuronal signaling. The conjugation and deconjugation of SUMO isoforms occur within minutes, allowing for rapid responses to changing cellular conditions. SUMO2/3 participates in early responses to stimuli such as oxidative stress, while SUMO1 is typically conjugated under more stable conditions due to its association with nuclear structures. SUMO2/3 can be conjugated almost immediately, underscoring its role in dynamic cellular responses. Also, the pool of free SUMO2/3 is greater than SUMO1 in all central nervous system regions.^44^^,^^45^ SUMO2/3 can form poly-SUMO chains via lysine 11 (K11), which serve as degradation signals for SUMO-targeted ubiquitin ligases. In contrast, SUMO1 is present primarily as a monomer, but it has been shown that SUMO1 can be linked to the end of a poly-SUMO2/3 chain and effectively terminate chain growth.^46^

The subcellular localization and temporal expression of SUMO ligases and the E2 enzyme Ubc9 modulate the efficiency of SUMO conjugation and its substrate preferences by recognizing consensus motifs. Although Ubc9 can recognize target motifs directly, many proteins require the presence of E3 ligases (*e.g.*, PIAS and RanBP2), which enhance specificity by providing additional interactions and facilitating the modification of less accessible lysine residues. SUMOylation also depends on the presence of SUMO-interacting motifs (SIMs) within target proteins.^47^^,^^48^ SIM-mediated interactions discriminate between SUMO paralogues by selectively stabilizing SUMO1 or SUMO2/3 bound complexes (non-covalent bindings). This allows for temporal coordination, for example, by promoting SUMO2/3 conjugation during early stress responses, followed by SUMO1-dependent modifications that support long-term maintenance of nuclear architecture.^49^

SENPs are key regulators of SUMOylation dynamics, providing precise temporal and spatial control of protein modifications within the cell. As mentioned above, they are responsible for the removal of SUMO target proteins, ensuring the reversibility and dynamics of the modification. Below are examples, well-documented in the literature, that demonstrate how various SENPs affect the temporal and spatial dynamics of the process.

SENP3 regulates the SUMOylation of the proline, glutamic acid, and leucine-rich protein 1 (PELP1) complex during pre-60S maturation. SENP1 and SENP2 influence the dynamics of chromatin-associated complexes by deSUMOylation of polycomb repressive complex 1 (PRC1). SENP6 restricts SUMO chain formation in response to DNA damage, synchronizing the recruitment of recombinase Rad51 to DNA lesion sites.

SENP1, SENP2, SENP3, and SENP7 operate within the nucleus (*e.g.*, on chromobox protein homolog 4 (CBX4)), controlling gene expression. SENP5 relocalizes to the mitochondrial surface during the G2/M transition, influencing mitochondrial division. SENP6 regulates the SUMOylation of replication protein A 70 kDa subunit (RPA70) within the chromatin during DNA replication and repair.

Control of SUMOylation by SENPs is a precise mechanism that allows the cell to dynamically respond to environmental changes, coordinate proliferative and immune signaling, and ensure genome integrity through finely tuned, temporal, and spatial modulation of key protein activities.^50^

### Crosstalk between SUMOylation and other PTMs

SUMOylation occurs in cooperation with other PTMs, such as phosphorylation and ubiquitination, and these processes are highly linked. For example, phosphorylation can either promote or inhibit SUMO conjugation via phosphorylation-dependent SUMOylation motifs (PDSMs, ΨKx(D/E)xxSP) or negative-dependent SUMOylation motifs (NDSMs, ΨKXEXXEEEE), where a serine residue adjacent to the SUMO consensus site must be phosphorylated to enable SUMO attachment (as observed in heat shock factors (HSFs), GATA binding protein 1 (GATA-1), and myocyte enhancer factor 2A (MEF2A)).^51^ This creates an additional layer of spatial and temporal regulation, especially under altered cellular conditions such as stress or during cell cycle transitions. Moreover, SUMOylation can itself regulate other PTMs by stabilizing or modulating the activity of kinases and phosphatases, thereby integrating complex signaling networks within the cell (*e.g.*, AKT).

SUMO-targeted ubiquitination is a process in which poly-SUMO2/3 chains are recognized by SUMO-targeted ubiquitin ligases, such as RING finger protein 4 (RNF4), which contains SIM domains that enable specific recognition of SUMOylated proteins. These ligases attach ubiquitin chains to SUMOylated substrates, thereby inducing their proteasomal degradation. This mechanism contributes to protein quality control by eliminating aberrant or excessively SUMOylated proteins and regulates various signaling pathways. Under conditions of oxidative stress, SUMO-targeted ubiquitin ligases facilitate the degradation of excessive SUMOylated proteins that may otherwise accumulate and disrupt cellular homeostasis. Therefore, maintaining a proper balance between SUMOylation, ubiquitylation, and phosphorylation is crucial, as disturbances in these PTMs can impair the regulation of signaling pathways responsible for sustaining cellular homeostasis.^52^

Taken together, these findings highlight that SUMOylation is a highly regulated and isoform-selective system. It operates with precise temporal and spatial control, determines substrate specificity, and integrates with other signaling pathways, allowing fine-tuning of cellular protein networks.

### Dysregulation of SUMOylation in GBM pathogenesis

Increasing evidence shows that the development of gliomas is complex and influenced by PTMs such as SUMOylation, which regulates the activity of thousands of proteins and is involved in numerous cellular processes.

Studies have demonstrated that enhanced SUMOylation increases the survival of GBM cells by protecting them from DNA damage, which may also contribute to therapy resistance through the promotion of double-strand break repair associated with increased levels of SUMO1 and SUMO2/3. Also, it is confirmed that the increased expression of the SUMO-specific E3 ligase, PIAS1, in GBM cells induces resistance to radiation-induced cell death by increasing the stress-inducible phosphoprotein STI1, leading to its nuclear accumulation.^15^

SUMOylation-induced modification of cyclins and cyclin-dependent kinases (CDKs) impacts the cell cycle. The mechanism by which SUMOylation affects CDK6 will be discussed later in this paper.

Moreover, analysis revealed that in GBM, SUMOylation also affects cellular metabolism, increasing the activity of glycolysis and the pentose-phosphate pathway, which is consistent with earlier reports on the role of SUMOylation in metabolism and the bioenergetic phenotype of GBM. This is likely due to the effect of SUMOylation on the hypoxia-inducible factor-1α (HIF-1α). Importantly, the stabilizing effect of SUMOylation on HIF-1α may also have implications for GBM progression via its influence on epithelial–mesenchymal transition. HIF-1α promotes the expression of epithelial-mesenchymal transition-associated proteins, and in the case of GBM, epithelial–mesenchymal transition is linked to tumor progression and the acquisition of a highly invasive phenotype.^15^ Assessing SUMOylation status may therefore be helpful in prognosis and in the future development of personalized therapies for patients with GBM.

## SUMO-affected molecular pathways, cell cycle checkpoints, and other molecular targets

### Cell cycle

The progression of the cell cycle is governed by a complex regulatory network ensuring the accurate transmission of genetic information. This process relies on precise coordination between various molecular factors that modulate transitions between different phases. A crucial aspect of cell cycle control is the presence of checkpoints that assess cellular conditions and detect potential errors. These checkpoints act as surveillance mechanisms, allowing for DNA damage repair before proceeding to the next stage. In cases where genomic integrity cannot be restored, the system activates programmed cell death (apoptosis) to prevent the propagation of defective cells.^53^ The proper functioning of the cell cycle is strictly dependent on cyclins, CDKs, and cyclin-dependent kinase inhibitors (CDKIs). Cyclins control CDKs and their substrate activity. The activity of the CDK/cyclin complex is tightly regulated by CDKIs.^37^

The progression of the cell cycle is controlled by a highly coordinated regulatory network that ensures the proper genomic transmission during cell division. This process depends on the temporal and spatial coordination of numerous molecular effectors that drive transitions between distinct cell cycle phases. A fundamental feature of cell cycle regulation is the existence of multiple surveillance checkpoints that monitor cellular status and detect genomic or mitotic abnormalities. These checkpoints, such as the G1/S and G2/M transitions, function as molecular control points that prevent the continuation of the cell cycle in the presence of DNA damage or incomplete replication. This enables the activation of DNA repair pathways and, if genomic integrity cannot be reestablished, initiates apoptosis to eliminate potentially oncogenic or dysfunctional cells.^53^

The proper functioning of the cell cycle machinery relies on the interplay between cyclins, CDKs, and CDKIs. Cyclins serve as regulatory subunits that bind to and activate CDKs, thereby directing kinase activity toward specific substrates required for phase-specific cellular events. The activity of CDK/cyclin complexes is controlled by CDKIs, which function as negative regulators to prevent unscheduled or excessive proliferation.^37^ Dysregulation of this balanced system is a hallmark of many cancers and contributes to unchecked cellular proliferation, genomic instability, and resistance to therapy. Particularly in GBM, alterations in the expression or activity of key CDKs, cyclins, and CDKIs have been implicated in tumor progression and therapeutic resistance. Therapeutic strategies targeting CDKs, either directly through selective inhibitors or indirectly by modulating upstream regulators such as SUMOylation pathways, represent a promising approach to restore cell cycle control in tumor cells.

Studies suggest that in humans, as in other organisms, SUMO proteins are concentrated at centromeres, kinetochores, and mitotic and meiotic chromosomes.^54^ The process of SUMOylation is essential for the proper segregation of chromatids during mitosis and for the regulated transition from metaphase to anaphase, as evidenced by live-cell imaging studies and analyses of chromatin bridge formation. The process of SUMOylation of SUMO target proteins, which are key factors for the proper functioning of the cell cycle, must occur in an accurate and orderly manner. This occurs through changes in the activity and localization of SUMO ligases and proteases. During mitosis, numerous SUMO ligases and proteases are then redistributed. The studies conducted proved that inhibition of SUMO-activating enzyme and SUMO-conjugating enzyme caused delayed mitosis and serious chromosomal aberrations, which may intensify the process of carcinogenesis.^15^^,^^55^

**Figure 2:**
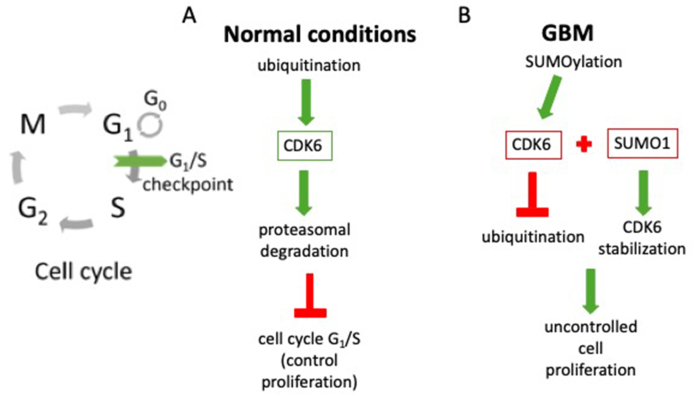
Cell cycle progression in normal cells versus glioblastoma cells. **(A)** Cell cycle progression in patients under normal conditions. The vertical dashes indicate inhibition, and the arrows indicate the flow of biological interactions. Under normal conditions, CDK6 undergoes ubiquitination and subsequent proteasomal degradation, leading to controlled cell cycle arrest at the G_1_/S checkpoint. **(B)** Cell cycle progression in patients with gliomas. The vertical dashes indicate inhibition, and the arrows indicate the flow of biological interactions. In glioblastoma (GBM), enhanced SUMOylation occurs, with SUMO1 binding to CDK6 and stabilizing it. This prevents CDK6 from undergoing ubiquitination and proteasomal degradation, ultimately leading to uncontrolled proliferation of GBM cells.

Nowadays, SUMOylation has been proven to be associated with many diseases, but the exact pathways of action are still not known. A study by Bellail et al indicated an association between SUMOylation and the cell cycle through stabilization of the CDK6 factor. Consequently, this caused glioma progression. CDK6 is a key regulator of the cell cycle, undergoing both SUMOylation and ubiquitination. However, conjugation of SUMO1 protects it from ubiquitin-dependent degradation. This occurs by stabilizing its protein levels and kinase activity (Fig. 2A). In GBM cells, SUMOylation of CDK6, which is initiated in the M phase by CDK1-dependent phosphorylation of Ubc9, is maintained into the G1 phase, subsequently driving the G1/S transition. In addition to the amplification of the gene itself and overexpression, the above mechanism seems to be the cause of the observed high levels of CDK6 in GBM. The cited studies clearly suggest that increased SUMO in GBM affects the cell cycle, ultimately leading to uncontrolled tumor proliferation (Fig. 2B).^15^^,^^37^ Recent studies have also helped to identify additional CDKs and cyclin molecules as SUMO targets. These include CDK1, CDK9, CDK11, and cyclin-E. These observations further emphasize the interplay between SUMO and phosphorylation processes during the regulation of the cell cycle.^56^^,^^57^

### The crosstalk between AKT signaling pathway and SAE1/2

AKT is a serine/threonine protein kinase that belongs to signaling molecules involved in a large number of biological processes. It can be activated through mechanisms such as SUMOylation, ubiquitination, and phosphorylation, affecting cell growth, differentiation, proliferation, and migration, as well as tumorigenesis.^58^^,^^59^ The major AKT SUMO acceptor is Lys276, and the phenomenon of SUMOylation is dependent on AKT phosphorylation status. It stabilizes AKT and increases its kinase activity, but does not affect the phosphorylation status.^60^ Activated AKT can alter the function of various downstream proteins, leading to changes in proliferation, migration, metabolism, and angiogenesis, thus inevitably increasing tumorigenic processes and significantly worsening patients' prognosis. The pro-oncogenic role of AKT was reported in various cancers, such as ovarian,^61^ breast, and prostate cancer,^62^ as well as gliomas.^23^ In studies conducted on pediatric patients with malignant gliomas, increased AKT protein expression was observed in 42 out of 53 tumors. It was also indicated that patients with lower AKT protein expression levels in tumor tissue exhibited a higher overall survival rate.^63^ A study conducted in 2021 also demonstrated a similar correlation between phosphorylated AKT protein levels in tumor tissue and survival of patients with gliomas, indicating that the activation of AKT-related signaling pathways impacts glioma patient outcomes.^64^

The proper ratio between SAE1 and SAE2 is essential for the correct progression of the SUMOylation process, and its dysregulation has a direct impact on the SUMOylation status of AKT, thereby modulating its stability, activity, and oncogenic potential in gliomas.^23^^,^^65^ Yang et al investigated the correlation between the expression of SAE1, activation of AKT, and human glioma progression. They revealed that high expression of SAE1 was correlated with an increase in SUMOylation and phosphorylation of AKT kinase, which leads to glioma cell growth.^23^ Yang et al also indicated that high expression of SAE1 was correlated with high-grade gliomas (grade III, IV) and low expression of SAE1 was associated with low-grade gliomas (grade I-II). They also mention the correlation between SAE1 and the cell cycle. The key point of their experiment was blocking SAE1 expression using siRNA treatment, which induced cell cycle arrest at the G2 phase and an elevation in apoptotic cell number.^23^ It should be noted that SAE1 inhibition led to changes in the expression of certain molecules associated with the cell cycle (CDK2, cyclin D1, and p21) and apoptosis (Bcl2 and active caspase-3). The mechanism of SAE1-induced glioma progression is based on SAE1 overexpression, which leads to increased SUMOylation and Ser473 phosphorylation of AKT. This, in turn, elevates the expression of CDK2, cyclin D1, and Bcl-2, factors responsible for phosphorylating proteins that regulate the G1/S phase transition (CDK2 and cyclin D1) and inhibiting apoptosis (Bcl-2), allowing tumor cells to continue dividing despite DNA damage signals (*e.g.*, temozolomide treatment). In this context, high CDK2 activity promotes entry into the S phase and progression of the cell cycle, overexpression of cyclin D1 accelerates the G1/S transition, and Bcl-2 promotes survival and proliferation of cancer cells. Due to accelerated DNA repair, excessive progression induces chemoresistance. This provides a clear picture of how SUMOylation and AKT phosphorylation drive the progression of tumors, including GBM, and induce a potential chemoresistance pattern.^23^ Silencing of SAE1 resulted in down-regulation of Bcl-2, p-AKT, CDK2, and cyclin D1. On the other hand, p21 and active caspase-3 expression were increased.^23^

Phosphorylation and SUMOylation of AKT are closely linked through specific motifs and regulatory mechanisms. The key elements for phosphorylation-driven SUMOylation are PDSMs, which enable SUMO binding via the negative charge of phosphorylated residues. Although AKT does not contain these consensus sequences directly, it can enhance its SUMOylation by phosphorylating SUMOylation enzymes: UBC9 (Thr35) and SUMO1 (Thr76). This creates a positive feedback loop that induces AKT SUMOylation and impacts other proteins (*e.g.*, PTEN). In some cases, phosphorylation can also inhibit SUMOylation by blocking binding to Ubc9 (*e.g.*, p53) or altering protein conformation, making it more susceptible to SUMO proteases. These interplay mechanisms between phosphorylation and SUMOylation play a critical role in regulating tumorigenic processes, including the progression of breast cancer or glioma.^66^

### Ubc9 and its regulative molecular pathways as potential therapy targets

Ubc9 protein (encoded by the *UBE2I* gene) is the only SUMO-conjugating enzyme with a crucial role in the SUMOylation catalytic cycle. Ubc9 plays a significant role in cell cycle progression, stress response, and DNA repair.^67^ Ubc9 overexpression has been linked to the progression of various cancers, such as cervical cancer,^61^ epithelial ovarian cancer,^61^ and prostate gland cancer.^68^

In the studies conducted by Zhao et al, they showed that the level of Ubc9 in both glioma tissue and glioma cell line was higher than the control tissue and cell line. After silencing Ubc9 expression with small interfering RNA (siRNA), inhibition of the growth of transfected glioma cells and promotion of their apoptosis were observed. An important aspect of the study was identifying the mechanism responsible for changes in E2 enzyme expression. Bioinformatics analysis revealed a statistically significant decrease in miR-212 expression in glioma tissues compared with normal brain tissues. These findings were experimentally confirmed in patient tissue samples. Additionally, overexpression of miR-214 in glioma cell lines inhibited translation of UBE2I mRNA, leading to a significant reduction in Ubc9 protein levels, while its effect on mRNA was minimal. Overexpression of miR-214 in glioma cells resulted in reduced tumor cell growth and significantly increased apoptosis. This may indicate the role of Ubc9 dysregulation in carcinogenesis, including GBM.^69^ Wang et al showed that overexpression of Ubc9 was involved in GBM cell proliferation, via collapsin response mediator protein (CRMP), which is a microtubule-associated protein involved in facilitation of neurite outgrowth, establishment of neuronal polarity, regulation of neurotransmitter release, and neuronal death.

This study showed that the cell line with up-regulated Ubc9 expression induced increased CRMP SUMOylation, and this influenced the increased GBM cell proliferation ratio in this cell line. This was confirmed by the fact that a mutation at K374A CRMP (region of SUMOylation) led to reduced SUMOylation of this protein and inhibition of cell proliferation.^70^ In the studies of Guo et al, it was shown that, in hypoxic conditions, there was an increase in Ubc9 expression, inducing the promotion of SUMOylation of hnRNP A2/B1 and its transfer to the cytoplasm of GBM cells. This, in turn, contributes to the sorting of miRNA 204-3p in exosomes and their transport to the cells of the tumor microenvironment, promoting angiogenesis and migration of tumor cells. The important role of SUMOylation in this process may be indicated by the fact that without its occurrence, there comes to overexpression of miRNA 204-3p (with an anti-cancer character) in GBM cells themselves, because it inhibited proliferation and prolonged overall survival, probably by inhibiting the cell cycle and the calcium voltage-gated channel subunit alpha-1C (CACNA1C)/mitogen-activated protein kinase (MAPK) pathway in GBM cells.^36^

In glioma stem cells, the increased expression of peptidyl-prolyl isomerase NIMA-interacting 1 (PIN-1) has been reported, which induces cancer cell progression. The Pin-1 isomerizes Ubc9 to facilitate formation of the Ubc9-SUMO1 thioester. Notably, Ubc9 contains one motif, which may be subject to Pin-1-catalyzed isomerization, composed of serine 71 (S71) and proline 72 (P72). The analyses by coimmunoprecipitation detected a strong interaction between Ubc9 and Pin-1, which may be summarized that Ubc9 is a substrate of Pin-1. Interestingly, despite Pin-1's ability to isomerize Ubc9, this modification does not appear to influence changes in its expression levels. The overexpression of Pin-1 is accompanied by an increased formation of the SUMO1-Ubc9 complex. These findings indicate an enhancement of global protein SUMOylation mediated by SUMO1 in glioma stem cells. It is widely known that enhanced SUMOylation is responsible for excessive proliferation. Blocking Pin-1 can slow down glioma progression by affecting Ubc9.^71^

This study further underscores the importance of researching SUMOylation and the individual molecules involved in this process. Once again, the critical role of SUMOylation in the development of gliomas and other cancers has been demonstrated. Investigating the mechanisms underlying SUMOylation dysfunction could play a pivotal role in developing modern therapeutic strategies for various diseases.

## Drugs with effects on SUMOylation

As SUMOylation is a global molecular process that exerts biological functions through post-translationally modified downstream targets, including those engaged in cell cycle regulation checkpoints, some effort has been put into describing the effects of various molecular agents on SUMO proteins and SUMOylation-related targets.^72^^,^^73^ We collected several papers regarding popular drugs and natural compounds with an effect on SUMO in cancer in Table 2. Regarding GBM research, topotecan is a well-established anti-cancer therapeutic agent with a mechanism potentially affecting SUMOylation in several glioma cell lines. In terms of natural compounds, chlorogenic acid may be a potential SUMO1-affecting prospect in glioma.^74^^,^^75^

**Table 2:** Selected drugs and natural compounds with effects on SUMOylation in cancer cells and animal models.

Drug/natural compound	Supposed effect on SUMOylation	Affected biological functions	Other affected targets/pathways	Investigated model	Reference
Topotecan	Global SUMOylation inhibition; SUMO1-CDK6 conjugation inhibition	Induces cell cycle arrest at G1/S phase	CDK6 (down), HIF-1α (down)	Mainly glioma U251, LN229, and Mz18 cells	Bernstock et al^74^
Chlorogenic acid	Induction of SUMO1 expression and stability; promoting SUMO1-dependent c-Myc SUMOylation which rescues p21 from the inhibitory effect of miR-17 family members	Hepatoma and lung cancer cells: inhibits proliferation, migration, and invasion; induces cell cycle arrest at the G_0_/G_1_ phase; Glioma cells: inhibits metabolism in mitochondria; inhibits invasion and migration; inhibits sphere formation *in vivo:* inhibits tumor growth	Hepatoma and lung cancer cells: Cell differentiation genes: KHSRP (up), p53 (up), and p21 (up) Poor cell differentiation genes: c-Myc (down) and CD44 (down) miR-17 family members: miR-20a (down), miR-93 (down), and miR-106b (down) Glioma cells: p-c-Myc (down), p21 (up), Tuj1 (up), and GFAP (up) *In vivo:* c-Myc (down), p-c-Myc (down), EPCAM (down), CD44 (down), p21 (up), miR-20a (down), miR-93 (down), and miR-106b (down)	Mainly hepatoma Huh7 and lung cancer H446 cells; and glioma U87MG and M059J cells (for other investigated cells, see the original reference); Huh7 hepatoma and H446 lung cancer xenografted mice	Huang et al^75^
Ginkgolic acid	Reduction of overall protein SUMOylation; inhibition of SUMO1-dependent IGF-1R SUMOylation, which presumably inhibits IGF-1R nuclear accumulation	Inhibits proliferation, migration, and epithelial-to-mesenchymal transition; inhibits tumor growth	E-cadherin (up), vimentin (down), MMP2 (down), nuclear IGF-1R (down), and SNAI2 (down)	Gastric cancer BGC823 and HGC27 cells; gastric cancer xenografted mice	Liu et al^76^
	Inhibition of SUMO1-dependent NEMO SUMOylation	Inhibits migration	NF-κB (down), IκBα (up), uPA (down), PAI-1 (down), CXCR4 (down), and MMP9 (down)	Breast cancer MCF-7 and MDA-MB-231 cells; reporter cell line HEK Blue Null 1; *in vitro* SUMOylation assay	Hamdoun et al^77^
Dexamethasone	Reduction of SUMO1 conjugates level; presumable inhibition of SUMO1-dependent HIF-1α and Oct4 SUMOylation, which suppresses their translocation to the nucleus	Induces cell adhesion and aging; induces cell damage; inhibits migration and invasion; inhibits neovascularization; increases chemosensitivity to HSVtk/GCV treatment; inhibits tumor growth; induces apoptosis	SOX2 (down), Oct4- (down), vimentin (down), E-cadherin (up), and HIF-1α (down)	CD133^+^/CD44^+^ hepatocellular carcinoma Hep3B-derived stem cells (*bone marrow-derived mesenchymal stem cells*); hepatocellular carcinoma xenografted mice	Jiang et al^78^
Melatonin	Inhibition of SUMO1 expression, which presumably blocks the nuclear translocation of nestin and its subsequent effect on c-Myc	Inhibits sphere formation; inhibits self-renewal; induces G2/M arrest; induces paclitaxel sensitivity	CDK2 (down), CDK4 (down), cyclin D1 (down), cyclin E1 (down), CXC4 (down), A20 (down), SOX2 (down), CD133 (down), NANOG (down), cyclin B1 (up), MT1 (up), c-Myc (down), nestin (down), c-Myc/nestin-related genes, H3K4me3 (down), and H3K36me3 (down)	X02 glioblastoma cells	Lee et al^79^
Fisetin	Direct binding of fisetin to conserved amino acid residues (L65, F66, E67, M82) of SUMO1 results in destabilization and unfolding of it	Inhibits p53 SUMOlation and thus enhances its proapoptotic function		*E. coli* Rosetta (DE3) line (competent); recombinant SMT3–HSF1 protein; SUMOlation enzyme systems	Velazhahan et al^80^
Luteolin	SUMO1 leads to the SUMOylation of lysines 480 and 585 of the ATP-binding domain of SERCA2a protein	Essential for maintaining SERCA2a stability and activity, thus contributing to the reduction of cardiomyocyte failure		Left ventricular samples of human heart from cardiac transplantation and from donors; wild-type and SUMO1-transgenic mice; rAAV and lentivirus vectors	Kho et al^81^
Curcumin	Return of SUMO1 and UBC9 concentrations to physiological values	Inhibits hyperactivation of p-JNK-SUMO1 axis; SUMO1 relocalizes from the perinuclear membrane to the cytoplasm and dispersion in the nucleus; prevent oxidative stress-related cell damage	Decrease in p-JNK/JNK, p-ERK/ERK, p-Tau/Tau ratio, Tau phosphorylation, and caspase-3 cleavage	SHSY5Y cell line	Buccarello et al^82^

Note: KHSRP, KH-type splicing regulatory protein; p-c-Myc, phosphorylated c-Myc; Tuj1, neuron-specific class III β-tubulin; GFAP, glial fibrillary acidic protein; EPCAM, epithelial cell adhesion molecule; IGF-1R, insulin-like growth factor 1 receptor; NEMO, NF-κB essential modulator; MMP2/9, matrix metalloproteinase 2/9; SNAI2, snail family transcriptional repressor 2 (also known as Slug); IκBα, inhibitor of nuclear factor kappa B alpha; uPA, urokinase-type plasminogen activator; PAI-1, plasminogen activator inhibitor-1; CXCR4, C-X-C motif chemokine receptor 4; Oct4, octamer-binding transcription factor 4; SOX2, SRY-box transcription factor 2; NANOG, homeobox protein NANOG; MT1, metallothionein 1; nestin, neuroepithelial stem cell protein; H3K4me3, trimethylation of lysine 4 on histone H3; H3K36me3, trimethylation of lysine 36 on histone H3; HSVtk/GCV treatment, herpes simplex virus thymidine kinase/ganciclovir Treatment; CD44, Cluster of Differentiation 44; CD133 (Prominin-1), Transmembrane glycoprotein marker for cancer stem cells; ERCA2a, sarcoplasmic/endoplasmic reticulum Ca^2+^-ATPase; JNK, c-Jun N-terminal kinase; p-JNK, phosphorylated c-Jun N-terminal kinase; ERK, extracellular signal-regulated kinase; p-ERK, phosphorylated extracellular signal-regulated kinase; TAU, microtubule associated protein tau; p-TAU, phosphorylated microtubule associated protein tau.

### Topotecan disrupts the cell cycle by inhibiting SUMOylation and CDK6 axis

Topotecan is a chemotherapeutic agent approved by the US Food and Drug Administration, with established clinical efficacy in the treatment of ovarian cancer and small-cell lung cancer. Its primary mechanism of action involves the inhibition of topoisomerase I, an enzyme essential for relieving torsional stress during DNA replication. By stabilizing the covalent DNA-topoisomerase I cleavage complex, topotecan induces DNA single-strand breaks, ultimately leading to replication fork collapse, genomic instability, and apoptotic cell death. This mechanism underlies its effectiveness in targeting rapidly proliferating tumor cells.^83^

A study conducted by Bernstock et al^74^ using human GBM cell lines revealed that topotecan treatment led to a reduction in the global levels of SUMO1- and SUMO2/3-conjugated proteins. Interestingly, this effect occurred without significant alterations in the expression of the E1 activating enzyme complex (SAE1/SAE2) or the E2 conjugating enzyme Ubc9, which are typically essential for canonical SUMOylation. This observation suggests that topotecan exerts a non-canonical, E1/E2-independent effect on the SUMOylation process.

Furthermore, Bernstock et al^74^ noted decrease in CDK6 protein levels upon topotecan treatment. This finding is particularly relevant in light of the work by Bellail et al^37^ that demonstrated that CDK6 was stabilized through SUMOylation, thereby linking the SUMOylation machinery to cell cycle regulation. To explore this relationship further, the authors assessed the level of SUMO1-conjugated CDK6 in topotecan-treated U251 glioma cells. They observed a marked reduction in CDK6-SUMO1 conjugates, confirming that topotecan disrupts CDK6 stability via suppression of SUMO1-dependent PTMs.^74^

Thus, the anti-tumor activity of topotecan extends beyond its established role as a topoisomerase I inhibitor. It also involves interference with the SUMOylation machinery, particularly through attenuation of the SUMO1-CDK6 axis, and appears to act through an E1/E2-independent pathway. These findings raise the possibility that topotecan may function as a dual-acting agent, exerting both genotoxic and post-translational regulatory effects in glioma cells. Given the role of CDK6 in promoting G1-S transition and glioma stem cell maintenance, its destabilization may represent an important therapeutic vulnerability in GBM. Moreover, interference with SUMOylation pathways may increase the susceptibility of tumor cells to additional stressors or therapeutic agents, providing a rationale for potential combinatorial approaches.

### Chlorogenic acid as a potential adjuvant therapy in cancer treatment

Chlorogenic acid, a natural compound present in coffee and green tea, is widely recognized for its anti-bacterial and anti-inflammatory properties. Recent studies indicate that chlorogenic acid may play a role in inhibiting cancer cell proliferation, although the precise molecular mechanisms underlying this effect warrant further elucidation.^84^ Some putative explanations of metabolic changes exerted on cancer cells by chlorogenic acid include its effects on AMP-activated protein kinase (AMPK), hypoxia-inducible factor 1 alpha (HIF-1α), and MAPK/extracellular signal-regulated kinase (ERK) pathways.^85^ Chlorogenic acid could induce apoptosis; it decreased the proliferation of lung cancer cells and viability of colorectal cancer cells and hampered the expression of apoptosis inhibitor BCL2^86^^,^^87^. In breast cancer cells, chlorogenic acid promoted transition from mesenchymal to epithelial phenotype and inhibited tumor growth in a xenograft mouse model.^88^ In another work regarding breast cancer, the apoptotic activity of chlorogenic acid, along with epithelial–mesenchymal transition inhibition and a decline in the levels of nuclear factor-kappa B (NF-κB)-related proteins, was observed. Further *in vitro* experiments confirmed chlorogenic acid-induced anti-cancer activity in a tumor-bearing mouse model.^88^

The study conducted by Huang et al^75^ was performed on many chlorogenic acid-treated tumor cell lines, including human hepatoma, lung cancer, and glioma, and aimed to examine chlorogenic acid activity as a cell differentiation agent. *In vitro* analyses showed that in chlorogenic acid-treated cells, proliferation, migration, and ATP production declined while the cell cycle was arrested at the G0/G1 phase. On the molecular level, chlorogenic acid increased SUMO1 protein expression, leading to enhanced SUMOylation of *c-Myc* while simultaneously up-regulating p21 protein level. In glioma cells, silencing SUMO1 reversed the chlorogenic acid-induced effect on ATP production, which suggests that chlorogenic acid exerts its mechanism via SUMO1 in mitochondria. Moreover, expression analysis of differentiation-related genes in both *in vitro and in vivo* models confirmed that chlorogenic acid may indeed be a valuable prospect in inducing maturation phenotype changes in cancer cells.^75^

## Conclusion

SUMOylation is a catalytic and reversible process regulated by enzymes (E1, E2, and E3 ligases), ensuring proper cellular function. As one of the PTMs, its dysregulation has been linked to the development and progression of multiple diseases, including gliomas.

Based on the data presented regarding SUMOylation and cell cycle regulation in GBM, several potential diagnostic and therapeutic markers can be distinguished. SAE1 is a significant marker of GBM progression. High SAE1 expression correlates with more advanced glioma grades (III, IV), whereas lower expression is associated with lower grades (I, II). Inhibition of SAE1 leads to cell cycle arrest in the G2 phase and an increase in apoptotic cells. SAE1 regulation affects the levels of CDK2, cyclin D1, and p21, as well as key apoptotic proteins such as Bcl-2 and active caspase-3, indicating its crucial role in GBM pathogenesis.

CDK6 plays a key role in SUMOylation and GBM proliferation. CDK6 stabilized by SUMOylation does not undergo ubiquitination, leading to uncontrolled cell division. High expression of CDK6 is associated with an aggressive tumor phenotype. Blocking CDK6 SUMOylation via topotecan leads to its degradation, which may serve as an adjuvant therapeutic strategy.

Ubc9 is another essential therapeutic target in GBM. Its overexpression has been demonstrated in GBM and other cancers, and Ubc9 silencing results in the inhibition of glioma cell growth and increased apoptosis. Ubc9 regulates the SUMOylation of proteins such as CRMP and hnRNP A2/B1, impacting cell proliferation and tumor microenvironment interactions. Pin1 facilitates the formation of the Ubc9-SUMO1 complex, enhancing global protein SUMOylation and leading to excessive GBM cell proliferation.

SUMOylation of AKT plays a crucial role in regulating GBM aggressiveness. It stabilizes AKT activity, influencing proliferation, migration, and angiogenesis, while high AKT expression correlates with lower survival rates in GBM patients. An imbalance between SAE1 and SAE2 affects AKT SUMOylation, enhancing its oncogenic potential. Interfering with the SAE1/AKT pathway may provide a therapeutic approach.

Potential therapeutic agents affecting SUMOylation in glioma include topotecan and chlorogenic acid. Topotecan reduces SUMOylated SUMO1 and SUMO2/3 conjugates independently of E1 (SAE1/SAE2) and E2 (Ubc9) enzymes, leading to CDK6 degradation and cell cycle arrest. Chlorogenic acid induces c-Myc SUMOylation and increases p21 expression, resulting in cell cycle arrest at the G0/G1 phase. Although the abovementioned agents constitute promising prospects in glioma therapy research, the cited studies rely only on cell experiments and animal models. Additionally, considering that both topotecan and chlorogenic acid can exert multimodal and pleiotropic action in cancer cells, the relevance of SMUOylation as a target for glioma therapy remains elusive and warrants further preclinical evaluation.

Among the analyzed factors, Ubc9 appears to be the most promising marker for GBM due to its key role in SUMOylation regulation and its association with multiple oncogenic pathways. Its overexpression is linked to aggressive tumor progression, and its inhibition leads to reduced proliferation and increased GBM cell apoptosis. Furthermore, its role in Pin1 interaction and influence on global SUMOylation makes it a promising therapeutic target. Further research on Ubc9 may provide new therapeutic strategies.

## Future perspectives

The role of SUMOylation in GBM remains an area of growing interest, offering new possibilities for both understanding tumor biology and developing targeted therapies. Several future directions can be considered.

### Targeting SUMOylation for therapeutic intervention

Since SUMOylation plays a key role in GBM progression by stabilizing oncogenic proteins (*e.g.*, AKT, CDK6, and c-Myc) and promoting chemoresistance, inhibitors of SUMO-conjugating enzymes (*e.g.*, Ubc9 and SAE1/2) may serve as novel therapeutic options. Developing selective and clinically viable SUMOylation inhibitors could enhance treatment efficacy, especially in combination with existing therapies.

### Combination therapies for enhanced efficacy

Given that topotecan has been shown to reduce CDK6 SUMOylation and degradation, similar strategies could be explored to enhance the therapeutic effects of CDK inhibitors. Additionally, natural compounds like chlorogenic acid, which modulate SUMOylation pathways, may serve as adjuvant treatments to improve patient outcomes.

### Exploring SUMOylation as a biomarker

The expression levels of SUMO-related enzymes (*e.g.*, Ubc9 and SAE1/2) could be further investigated as potential prognostic and predictive biomarkers for GBM progression, therapy resistance, and patient survival. SUMOylation status may help stratify patients for personalized treatment approaches.

### Understanding the crosstalk between SUMOylation and other PTMs

SUMOylation interacts with other PTMs, such as ubiquitination and phosphorylation, influencing tumor cell survival and therapy resistance. Further research into these regulatory networks may uncover novel therapeutic vulnerabilities in GBM.

### Overcoming drug resistance through SUMOylation modulation

GBM is highly resistant to conventional treatments, including temozolomide and radiation therapy. Since SUMOylation contributes to DNA damage response and repair, targeting this pathway may enhance the sensitivity of glioma cells to existing treatments and prevent tumor recurrence.

### SUMOylation in glioma stem cells

Glioma stem cells drive tumor recurrence and resistance to therapy. Understanding how SUMOylation supports the maintenance and proliferation of glioma stem cells may reveal new approaches to eradicate these therapy-resistant cell populations.

In conclusion, targeting SUMOylation represents a promising but unexplored strategy in GBM treatment. Future studies should focus on developing specific inhibitors, identifying predictive biomarkers, and integrating SUMOylation-based therapies with existing treatment regimens to improve patient prognosis.

## CRediT authorship contribution statement

**Wiktoria Dubanosow:** Writing – review & editing, Writing – original draft, Conceptualization. **Bartosz Lenda:** Writing – review & editing, Writing – original draft, Conceptualization. **Marta Żebrowska-Nawrocka:** Writing – review & editing, Writing – original draft, Conceptualization. **Dagmara Szmajda-Krygier:** Writing – review & editing, Writing – original draft. **Rafał Świechowski:** Writing – review & editing, Writing – original draft. **Ewa Balcerczak:** Writing – review & editing, Writing – original draft, Supervision, Funding acquisition, Conceptualization.

## Funding

This present study was supported by statutory funds from the Department of Pharmaceutical Biochemistry and Molecular Diagnostics, Medical University of Lodz, Poland (No. 503/3-015-02/503-31-001).

## Conflict of interests

The authors have no competing interests to declare.
